# Functional annotation of HOT regions in the human genome: implications for human disease and cancer

**DOI:** 10.1038/srep11633

**Published:** 2015-06-26

**Authors:** Hao Li, Hebing Chen, Feng Liu, Chao Ren, Shengqi Wang, Xiaochen Bo, Wenjie Shu

**Affiliations:** 1 Department of Biotechnology, Beijing Institute of Radiation Medicine, Beijing 100850, China

## Abstract

Advances in genome-wide association studies (GWAS) and large-scale sequencing studies have resulted in an impressive and growing list of disease- and trait-associated genetic variants. Most studies have emphasised the discovery of genetic variation in coding sequences, however, the noncoding regulatory effects responsible for human disease and cancer biology have been substantially understudied. To better characterise the *cis*-regulatory effects of noncoding variation, we performed a comprehensive analysis of the genetic variants in HOT (high-occupancy target) regions, which are considered to be one of the most intriguing findings of recent large-scale sequencing studies. We observed that GWAS variants that map to HOT regions undergo a substantial net decrease and illustrate development-specific localisation during haematopoiesis. Additionally, genetic risk variants are disproportionally enriched in HOT regions compared with LOT (low-occupancy target) regions in both disease-relevant and cancer cells. Importantly, this enrichment is biased toward disease- or cancer-specific cell types. Furthermore, we observed that cancer cells generally acquire cancer-specific HOT regions at oncogenes through diverse mechanisms of cancer pathogenesis. Collectively, our findings demonstrate the key roles of HOT regions in human disease and cancer and represent a critical step toward further understanding disease biology, diagnosis, and therapy.

Because of advances in genome-wide association studies (GWAS)^1^,^2^ and on-going large-scale cancer genome sequencing projects^3^,^4^, an impressive list of disease- and trait-associated genetic variants has been produced, and the list continues to grow. To date, most studies spanning diverse diseases and quantitative phenotypes have focused on genetic variation in coding sequences. These studies have substantially extended our understanding of genetic variation in coding regions, which comprise less than 2% of the human genome. However, the role of genetic variations in noncoding regions in tumorigenesis have not been as extensively studied as the role of variations in coding regions, with the exception of some isolated examples^5^,^6^,^7^,^8^,^9^.

The recently completed Encyclopaedia of DNA Elements (ENCODE) project^10^,^11^ is by far the best known effort to identify functional elements in the human genome. This project has resulted in the striking conclusion that at least 20% of the genome possesses biological functions, whereas more than 80% exhibits biochemical functions^10^. Although variants in noncoding regions have been linked to diseases, their functional implications remain poorly understood^12^,^13^. In an effort to interpret the mechanistic roles of disease- and trait-associated genetic variants, recent studies have demonstrated that the vast majority of these variants are commonly located within regulatory DNA elements^14^,^15^. Thus, the recurrent noncoding mutations observed in human disease and cancer could have a regulatory effect. Recent reports have presented several lines of evidence concerning the roles of common noncoding DNA variants in transcriptional regulatory mechanisms, including the modulation of promoter and enhancer elements^16^,^17^,^18^,^19^ and enrichment within expression quantitative trait loci (eQTL)^19^,^20^,^21^. Although the functions of only a minority of these variants have been described, the development of sequencing technologies has enabled systematic whole-genome sequencing of large collections of samples capable of elucidating possible roles for noncoding mutations in the formation and development of human diseases and cancers.

HOT (high-occupancy target) regions are bound by a surprisingly large number of transcription factors (TFs) and are considered one of the most intriguing findings of recent large-scale sequencing studies, such as the ENCODE and modENCODE projects. These studies have demonstrated the widespread presence of HOT regions in worms^22^,^23^, flies^24^,^25^,^26^,^27^,^28^, and humans^29^,^30^,^31^, thus suggesting that HOT regions might reflect a general property of regulatory genomes. However, the functional significance of HOT regions is currently unclear^32^,^33^, as is the biological effect of these regions on human diseases and cancers. In our recent study^34^, we produced a comprehensive catalogue of transcription factor binding site (TFBS)-clustered regions in a broad range of human cell types and assigned a “TFBS complexity” score to each TFBS-clustered region. In a subsequent study, we performed an integrative analysis of one class of TFBS-clustered regions, HOT regions, which are defined as TFBS-clustered regions with extremely high TFBS complexity. We observed that HOT regions associate with genes that largely define the development and differentiation of the respective cell and tissue types (Li *et al*., in preparation). Our prior studies laid the foundation for a systematic annotation of the functional roles of HOT regions in the control of human diseases and cancers.

Here, we performed a comprehensive analysis of variants, especially noncoding variants, in HOT regions across diverse human cell types and tissues. We observed that GWAS SNPs that map to HOT regions undergo a substantial net decrease and demonstrate a cell- and development-specific localisation during development along the hematopoietic lineage. Additionally, HOT regions are disproportionally enriched for genetic variants compared with LOT (low-occupancy target) regions in both disease-relevant and cancer cells, and the enrichment is biased toward disease- or cancer-specific cell types. Furthermore, we demonstrated that cancer cells generally acquire cancer-specific HOT regions in oncogenes through diverse mechanisms during tumour pathogenesis. Finally, we discuss the implications of these findings for future investigations of human diseases and cancers.

## Results

### GWAS SNPs in development-specific HOT regions

**Figure 1:**
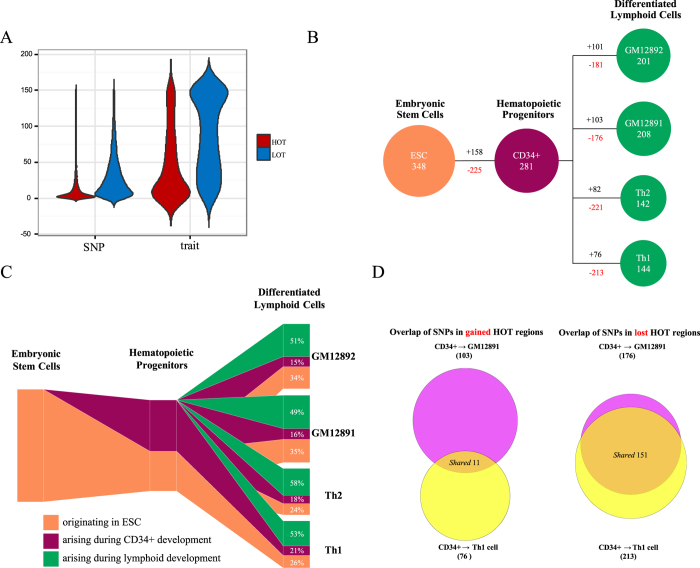
GWAS SNPs in development-specific HOT regions during haematopoiesis. (**A**) Distributions of the number of cell types, from 1 to 154 (*y* axis), in which SNPs and their corresponding phenotypic traits localised within HOT regions (red) and LOT regions (blue) (*x* axis) were observed. The width of each shape at a given *y* value indicates the relative frequency of SNPs and phenotypic traits within HOT and LOT regions in that number of cell types. (**B,C**) SNP composition of developing hematopoietic HOT regions. (**B**) Increased (black) versus decreased (red) numbers of SNPs mapping to HOT regions during hematopoietic developmental transitions are shown. (**C**) Schematic illustration of the number of SNPs that map to inherited versus acquired HOT regions during hematopoietic developmental transitions. The lymphoid HOT region compartment coloured orange constitutes a strict subset of the hematopoietic progenitor HOT region compartment. (**D**) Preferential extinction of common SNPs that map to HOT regions during development. Comparison of SNPs that map to acquired (left) or lost (right) HOT regions during differentiation of hematopoietic progenitors. See also Fig. S1 and Tables S1–S2.

Recent reports have suggested that many noncoding variants associated with common diseases and traits are concentrated in regulatory DNA regions marked by DNase I hypersensitive sites (DHSs)^14^,^35^. To investigate the extent to which disease-associated variation occurs in HOT and LOT regions, we compiled a list of 5,339 single-nucleotide polymorphisms (SNPs) linked to 529 diseases and phenotypic traits from 1,044 GWAS and investigated their distributions within HOT and LOT regions identified in 57 human cell and tissue samples (Table S1). The majority of GWAS SNPs (4,985, 93%) occurred in noncoding regions. Of these noncoding SNPs, 86% fell into the ~59% of the genome covered by all HOT and LOT regions (permutation test, *p* < 10^−4^) (Table S2). Additionally, 1,767 out of 4,985 SNPs (35%) fell into HOT regions (permutation test, *p* < 10^−4^), whereas 2,800 (48%) were in strong linkage disequilibrium (LD, *r*^2^ > 0.8) with SNPs in nearby HOT regions (Fig. S1A). Notably, the disease-associated SNPs and the corresponding phenotypic traits that mapped in HOT regions were generally more cell-specific than SNPs and traits in LOT regions, respectively (Fig. 1A). Moreover, the traits that mapped in both HOT and LOT regions were highly promiscuous compared with SNPs. Our results indicated that disease-associated variation and traits that map to HOT regions exhibits cell selectivity.

Our recent study demonstrated that HOT regions drive the expression of genes that largely define the development and differentiation of the respective cell and tissue types (Li *et al*., in preparation). However, how GWAS SNPs interact with developmentally dynamic (i.e., lost or gained) HOT regions remains poorly understood. Thus, we explored the dynamic changes in the genetic variation landscape during the transition from embryonic stem cells (ESCs) to hematopoietic progenitors and to T cells or B cells, given that development along the hematopoietic lineage has been extensively characterised at both the cellular and molecular levels^36^. We observed that during haematopoiesis, GWAS variants that map to HOT regions undergo a substantial net decrease (Fig. 1B,C). Comparison of ESC variants with those of their hematopoietic progenitors revealed a net decrease of 67 SNPs that map to HOT regions through the decrease of 225 ESC SNPs and the increase of 158 SNPs. Because hematopoietic progenitors terminally differentiate into B-lymphocytes or Th cells, they preferentially decrease a common set of 195 early developmental SNPs that map to HOT regions and increase an average of 90 chiefly lineage-restricted SNPs that map to HOT regions along each terminal branch (Fig. 1D). Of note, roughly half of the GWAS variants that map to the HOT regions of each definitive lymphoid cell type were arised during lymphoid development and roughly one-third were retained from the ESCs (Fig. 1C). Our results demonstrated that GWAS SNPs that map to HOT regions undergo a substantial net decrease during haematopoiesis.

**Figure 2:**
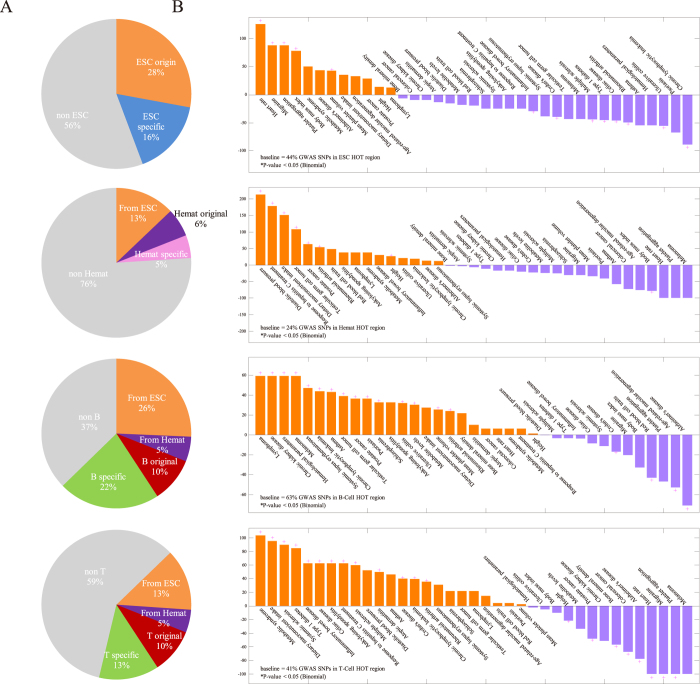
Enrichment of GWAS variants and phenotypes during haematopoiesis. (**A**) Proportion of GWAS SNPs selectively localised in HOT regions active along the hematopoietic lineage. ESC stage-specific HOT regions (blue); hematopoietic progenitor stage-specific HOT regions (pink); lymphoid cell stage-specific HOT regions (green); ESC-originated HOT regions (salmon); hematopoietic progenitor-originated HOT regions (purple); lymphoid cell-originated HOT regions (red) and HOT regions that belong to other stages (grey). (**B**) GWAS SNPs in HOT regions along the hematopoietic lineage exhibit phenotype-specific enrichment for development-specific HOT regions.

To further understand the dynamic interaction between GWAS variants and HOT regions during development and differentiation, we investigated whether HOT regions that harbour GWAS variants were active during the hematopoietic developmental stages (Fig. 2A). Out of 1,172 noncoding disease-associated SNPs located within global HOT regions, 44%, 24%, 63%, and 41% were located within HOT regions that were active in ESCs, hematopoietic progenitor cells, B cells, and T cells, respectively. A total of 28% of HOT regions that contain disease-associated variants were first detected in ESCs (“ESC origin” HOT regions), whereas 16% were in ESC-specific HOT regions. Similarly, 6% were first detected in hematopoietic progenitor cells (“hematopoietic origin” HOT regions), whereas 5% were in hematopoietic-specific HOT regions. A total of 10% were first detected in B cells (“B cell origin” HOT regions), whereas 22% were in B cell-specific HOT regions. Finally, 10% were first detected in T cells (“T cell origin” HOT regions), whereas 13% were in T cell-specific HOT regions.

Next, we analysed the enrichment or depletion of disease- and trait-specific GWAS variants located in HOT regions during hematopoietic development relative to the proportion of total GWAS SNPs in these HOT regions (Fig. 2B). In ESC HOT regions, we observed a significant enrichment in phenotypes for which growth trajectory and nervous system diseases have been demonstrated to play major roles, including platelet aggregation, body mass index, heart rate, Alzheimer’s disease, and migraines. In contrast, we observed differential relative depletion in ESC HOT regions associated with immune system diseases, including multiple sclerosis, Type 1 diabetes, rheumatoid arthritis, systemic lupus erythematosus, and digestive system diseases, such as Cohn’s disease, inflammatory bowel disease, and celiac disease. Immune system diseases, including rheumatoid arthritis and Kawasaki disease, and haematology-associated traits, including platelet counts, blood pressure, systolic blood pressure, haematology traits, and mean corpuscular haemoglobin, were significantly associated with hematopoietic HOT regions. In contrast, growth phenotypes such as body mass index were differentially depleted. In B and T cell HOT regions, we observed a significant association with immune system and digestive system diseases, which were significantly depleted in the ESC HOT region analysis. Additionally, immune-associated cancers, including chronic lymphocytic leukaemia, melanoma, and lymphoma, were significantly associated with these regions. In contrast, we observed differential relative depletion of nervous system diseases and growth trajectories, which were significantly associated with the ESC HOT regions. Together, these findings suggest that GWAS SNPs and phenotypes are enriched in a development-specific fashion during haematopoiesis.

### Disproportional enrichment of SNPs in HOT regions

**Figure 3:**
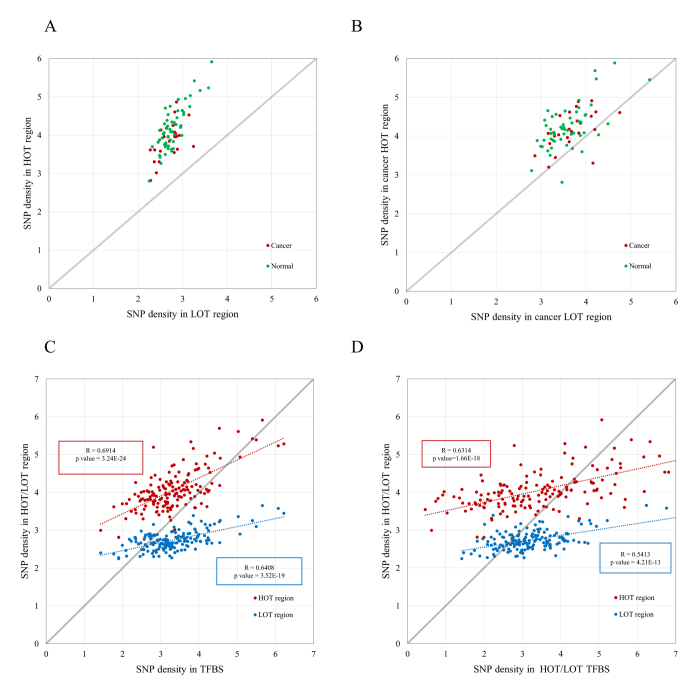
Disproportional enrichment of SNPs in HOT regions. (**A,B**) Scatter plot of the densities of GWAS SNPs (**A**) and SNPs in strong LD (*r*^2^ > 0.8) with GWAS SNPs (**B**) in HOT regions (*y* axis) and LOT regions (*x* axis) in 57 disease-relevant cells (green) and 25 cancer cells (red). (**E,F**) Scatter plot of the SNP densities in HOT/LOT regions (*y* axis) and SNP density (*x* axis) in genome-wide TFBSs in the human genome (**E**) and in TFBSs that map to HOT/LOT regions (**F**) across 154 ENCODE cell lines. The Pearson’s correlation coefficients and corresponding *p*-values are indicated. See also Tables S1–S2.

To gain further insight into the function of HOT regions in human diseases and cancers, we explored the association of genetic variants with HOT regions identified in both disease-relevant cells and cancer cells (Fig. 3). We observed that for both 57 disease-relevant cells and 25 cancer cells (Table S1), GWAS SNPs were significantly enriched in HOT and LOT regions (permutation test, *p* < 10^−4^). Notably, the enrichment was disproportionally in HOT regions compared with LOT regions in 57 disease-relevant cells and 25 cancer cells (Fig. 3A, χ^2^ test, χ^2^ = 172.1, *p*-value < 2.2 × 10^−16^ and χ^2^ = 125.2, *p*-value < 2.2 × 10^−16^, respectively). Furthermore, consistent results were obtained for SNPs in strong LD with GWAS SNPs (Fig. 3B, χ^2^ test, χ^2^ = 955.3, *p*-value < 2.2 × 10^−16^ and χ^2^ = 496.8, *p*-value < 2.2 × 10^−16^, respectively). For certain diseases and cancers, the disproportional enrichment in HOT regions was particularly striking (Fig. S1C). Our findings confirm that most disease-associated variants occur in regulatory DNA regions^14^,^35^ and reveal that HOT regions are disproportionally enriched for genetic risk variants compared with LOT regions in human cell and tissue types.

Because HOT regions are defined as TFBS-clustered regions with extremely high TFBS complexity^34^, it is reasonable to check whether the disproportional enrichment of HOT regions for genetic variants compared with LOT regions was caused by the extremely high concentration of TFBSs that map to HOT regions relative to LOT regions. For this purpose, we calculated the densities of GWAS SNPs within HOT regions, LOT regions, and genome-wide TFBSs in the human genome across 154 ENCODE cell lines (Fig. 3C). For a large majority of cell types (90%, 138 out of 154), the GWAS SNP densities in HOT regions are greater than those in TFBSs (above the diagonal), whereas the SNP densities in LOT regions are much less than those in TFBSs (below the diagonal). Furthermore, both the SNP densities in HOT (*R* = 0.6914, *p*-value = 3.24 × 10^−24^) and LOT (*R* = 0.6408, *p*-value = 3.52 × 10^−19^) regions exhibit significant correlations with SNP densities in genome-wide TFBSs in the human genome. To further elucidate the cause of this disproportional enrichment, we compared the SNP densities in HOT/LOT regions with the SNP densities in TFBSs that map to corresponding HOT/LOT regions (Fig. 3D). For nearly 70% of cell types (68%, 105 out of 154), the SNP densities in HOT/LOT regions are much higher/lower than those in the TFBSs that map to HOT/LOT regions, respectively. Both the correlations of SNP densities in HOT (*R* = 0.6314, *p*-value = 1.66 × 10^−18^) and LOT (*R* = 0.5413, *p*-value = 4.21 × 10^−13^) with SNP densities in TFBSs that map to HOT/LOT regions are still significant. These findings suggest that the SNP densities in TFBSs are significantly correlated with the SNP densities in HOT/LOT regions, but the high concentration of TFBSs alone cannot completely determine the disproportional enrichment of GWAS SNPs that map to HOT regions compared with LOT regions.

### GWAS SNPs in disease-specific HOT regions

**Figure 4:**
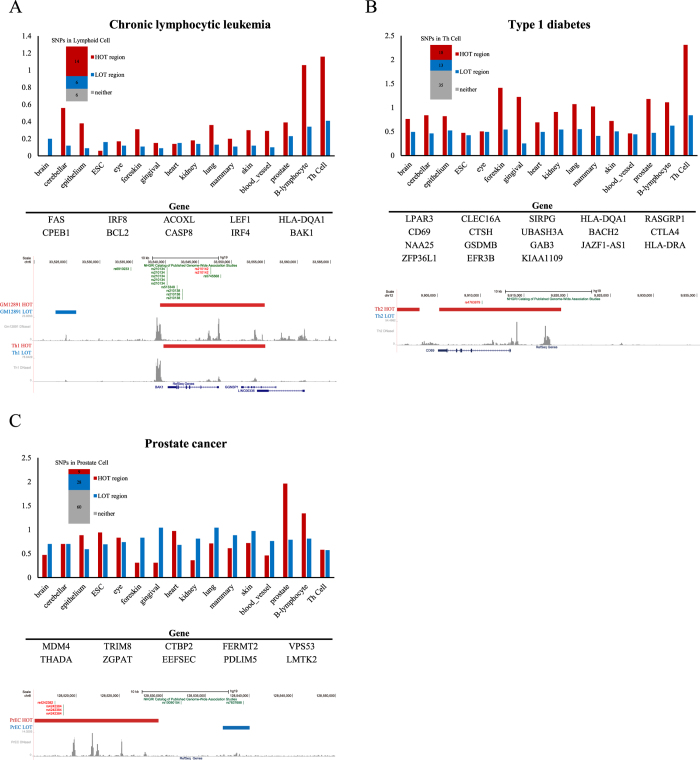
GWAS SNPs in disease-specific HOT regions. (**A**) (Upper) Bar plots that show the density (SNP/MB) of trait-associated noncoding SNPs linked to CLL in the HOT and LOT region domains identified in 16 human cell and tissue types. (Middle) List of genes associated with CLL SNP-containing HOT regions in T cells and B cells. (Bottom) DNase-seq profiles at the BAK1 locus in GM12891 and Th1 cells. The positions of the CLL SNPs are highlighted with red lines, the HOT regions are highlighted with red bars, and the LOT regions are highlighted with blue bars above the binding profile. (**B**) (Upper) Bar plots that show the density (SNP/MB) of trait-associated noncoding SNPs linked to type 1 diabetes (T1D) in the HOT and LOT region domains identified in 16 human cell and tissue types. (Middle) List of genes associated with T1D SNP-containing HOT regions in T cells. (Bottom) DNase-seq profile at the *CD69* locus in Th2 cell. The positions of the T1D SNPs are highlighted with red lines, the HOT regions are highlighted with red bars, and the LOT regions are highlighted with blue bars above the binding profile. (**C**) (Upper) Bar plots that show the density (SNP/MB) of trait-associated noncoding SNPs linked to prostate cancer in the HOT and LOT region domains identified in 16 human cell and tissue types. (Middle) List of genes associated with prostate cancer SNP-containing HOT regions in PrEC cell. (Bottom) DNase-seq profile in prostate epithelial cells. The positions of prostate cancer SNPs are highlighted with red lines, the HOT regions are highlighted with red bars, and the LOT regions are highlighted with blue bars above the binding profile. See also Fig. S2.

Because HOT regions are disproportionally enriched for disease-associated variants compared with LOT regions, we would expect that variants associated with specific diseases would tend to occur in the HOT regions of disease-relevant cell or tissue types, which are generally referred to as physiologically or pathogenically relevant cell or tissue types, including affected tissues and known or posited effector cell types^14^. Indeed, for a broad spectrum of diseases, we observed that disease-associated variants tended to occur in the HOT regions of disease-relevant cells and not in those of disease-irrelevant cells. This relationship was more pronounced for HOT regions than for LOT regions (see the distribution bars in Fig. 4 and S1). To gain further insight into the relationship between these variants, HOT regions, and their associated genes, we focused on several diseases in which variants occur in HOT regions in disease-relevant cells. The diseases that were selected for further study included chronic lymphocytic leukaemia, type 1 diabetes, and prostate cancer (Fig. 4).

Chronic lymphocytic leukaemia (CLL) is the most common form of leukaemia in adults in Western countries. CLL is a complex immunologic disease that originates from antigen-stimulated mature B lymphocytes^37^. The SNP catalogue contained 28 SNPs linked to chronic lymphocytic leukaemia, of which 26 occurred in noncoding sequences. The noncoding SNPs were particularly enriched in the HOT regions of hemangioblast derivatives, including B cells and Th cells; a total of 12 and 7 SNPs occurred in the HOT regions of genes with prominent roles in B cells and Th cells, respectively (Fig. 4A). Thus, 54% (14/26) of all of the chronic lymphocytic leukaemia SNPs located in noncoding regions occurred in the 5.3% and 3.1% of the genomic regions of B and Th cells encompassed by HOT regions, respectively (permutation test, *p* < 10^−4^). One SNP (rs210142) occurred in the HOT region associated with the gene *BAK1* (Fig. 4A), whose reduced expression has recently been demonstrated to influence the risk of CLL^38^.

Type 1 diabetes is a T cell-mediated autoimmune disease in which the insulin-producing pancreatic beta cells are selectively destroyed; this disease is most often diagnosed in children and young adults. Much of the genetic variation implicated in type 1 diabetes is associated with major histocompatibility antigens, interleukin-2 signalling, T cell receptor signalling, and interferon signalling^39^,^40^,^41^. The SNP catalogue contained 76 SNPs linked to type 1 diabetes, of which 66 occurred in noncoding sequences. The noncoding SNPs were particularly enriched in the HOT regions of Th cells, with 18 occurring in the HOT regions of genes with prominent roles in Th cell biology (Fig. 4B). Notably, 27% (18/66) of the type 1 diabetes SNPs located in noncoding regions occurred in the 3.1% of the genome encompassed by Th cell HOT regions (permutation test, *p* < 10^−4^). One SNP (rs4763879) occurred in the HOT region associated with the gene *CD69* (Fig. 4B), which has been demonstrated to be associated with Type 1 diabetes^42^,^43^.

Prostate cancer is the most commonly diagnosed malignancy and the second most common cause of cancer-related deaths affecting males in developed countries. In addition to age and race/ethnicity, family history has long been a well-established risk factor for prostate cancer^44^. To date, GWAS have identified more than 100 independent susceptibility loci associated with prostate cancer, which cumulatively account for approximately 33% of the familial risk for this disease^45^,^46^. Of the 99 SNPs linked to prostate cancer in the SNP catalogue, 97 SNPs occur in noncoding regions. The noncoding SNPs were particularly enriched in the HOT regions of prostate tissues, with 9 occurring in the HOT regions of genes with prominent roles in prostate tissue biology (Fig. 4C). SNP rs4242382, which is located at 8q24 in the distal HOT region in prostate tissue (Fig. 4C), was reported to be strongly associated with the risk of prostate cancer^47^.

Similar disproportional enrichment in disease-specific HOT regions was observed for many additional diseases and traits, including ventricular conductions, rheumatoid arthritis, and celiac disease (Fig. S2). Together with these examples, our results demonstrated that the disproportional enrichment of HOT regions for GWAS SNPs is biased toward disease-relevant cells.

### SNPs in strong LD with GWAS SNPs in disease-specific HOT regions

**Figure 5:**
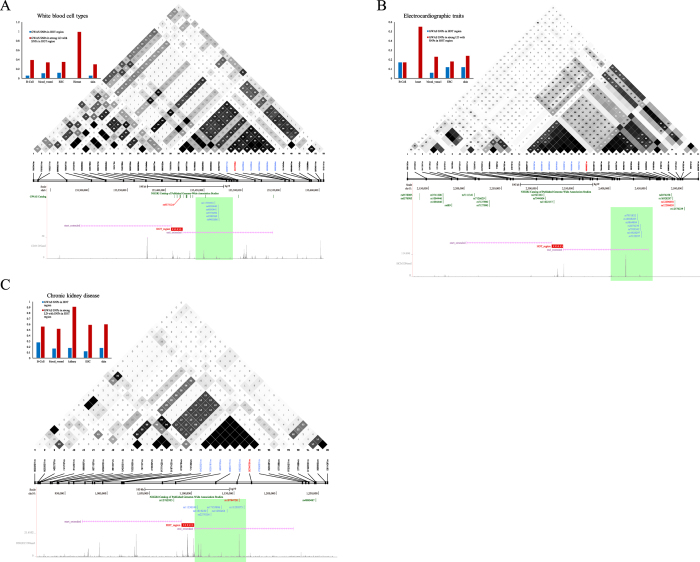
SNPs in strong LD with GWAS SNPs in disease-specific HOT regions. (**A**) (Upper-left) Bar plots that show the density of white blood cell type (WBCT)-associated noncoding SNPs and SNPs in strong LD (*r*^2^ > 0.8) with WBCT-associated SNPs (SNP/MB) in the HOT region domains and LOT region domains identified in 5 human cell and tissue types. (Upper-right) Example of GWAS SNPs in LD with WBCT-associated noncoding SNP rs9373124 (red bar). (Bottom) Distribution of GWAS SNPs (blue bar) in strong LD (*r*^2^ > 0.8) with rs9373124. The HOT region is extended by 250 kb. (**B**) (Upper-left) Bar plots that show the density of electrocardiographic trait-associated noncoding SNPs and SNPs in strong LD (*r*^2^ > 0.8) with electrocardiographic trait-associated SNPs (SNP/MB) in the HOT and LOT region domains identified in 5 human cell and tissue types. (Upper-right) Example GWAS SNPs in LD with electrocardiographic trait-associated noncoding SNP rs1296050 (red bar). (Bottom) Distribution of GWAS SNPs (blue bar) in strong LD (*r*^2^ > 0.8) with rs1296050. The HOT *r*egion is extended by 250 kb. (**C**) (Upper-left) Bar plots that show the density of chronic kidney disease (CKD) trait-associated noncoding SNPs and SNPs in strong LD (*r*^2^ > 0.8) with CKD trait-associated SNPs (SNP/MB) in the HOT and LOT region domains identified in 5 human cell and tissue types. (Upper-right) Example of GWAS SNPs in LD with CKD trait-associated noncoding SNP rs10794720 (red bar). (Bottom) Distribution of GWAS SNPs (blue bar) in strong LD (*r*^2^ > 0.8) with rs10794720. The HOT region is extended by 250 kb.

For some diseases or traits, a large proportion of GWAS SNPs are in strong LD (*r*^2^ > 0.8) with SNPs in a nearby HOT region, while only a few are located within that HOT region. To further understand the disproportional enrichment of HOT regions for SNPs in strong LD with GWAS SNPs and the bias of this enrichment toward disease-relevant cells, we focused on several diseases in which SNPs in strong LD with GWAS SNPs occur in HOT regions in disease-relevant cells (Fig. 5). White blood cells are blood cells that mediate immunity and play essential roles in defending the body against foreign microorganisms^48^. The SNP catalogue contains 20 SNPs linked to white blood cell types that occur in noncoding sequences. None of these SNPs are located in the HOT regions of the hematopoietic progenitor cells (0/20). However, a collective 30% (6/20) enrichment of GWAS SNPs are in strong LD (*r*^2^ > 0.8) with SNPs in the 1.8% of the genome sequences encompassed by hematopoietic progenitor HOT regions (permutation test, *p* < 10^−4^) (Fig. 5A).

Electrocardiographic traits are important, substantially heritable determinants of the risk of arrhythmias and sudden cardiac death^49^. The SNP catalogue contains 21 SNPs linked to electrocardiographic traits, 17 of which occur in noncoding sequences. None of these 17 GWAS SNPs are located within heart tissue HOT regions; however, 4 (24%) are in strong LD (*r*^2^ > 0.8) with SNPs in nearby heart tissue HOT regions, covering 2.1% of the genome sequences (permutation test, *p* < 10^−4^) (Fig. 5B).

Chronic kidney disease (CKD) is a progressive disorder that results in decreasing kidney function over a period of months to years. CKD has been increasingly recognised as a global public health problem^50^. Recent genetic studies have identified common CKD susceptibility variants that are associated with kidney function in European and African-American populations^51^,^52^,^53^,^54^,^55^. The SNP catalogue contains 30 SNPs linked to electrocardiographic traits, 27 of which occur in noncoding sequences. Only 1 GWAS SNP lies within a kidney tissue HOT region; however, 5 (19%) are in complete LD (*r*^2^ > 0.8), with SNPs in nearby kidney tissue HOT regions covering 2.2% of the genome sequences (permutation test, *p* < 10^−4^) (Fig. 5C). We further determined that the disproportional enrichment of HOT regions for SNPs in strong LD with GWAS SNPs is biased toward disease-relevant cells.

### SNPs in cancer-specific HOT regions

**Figure 6:**
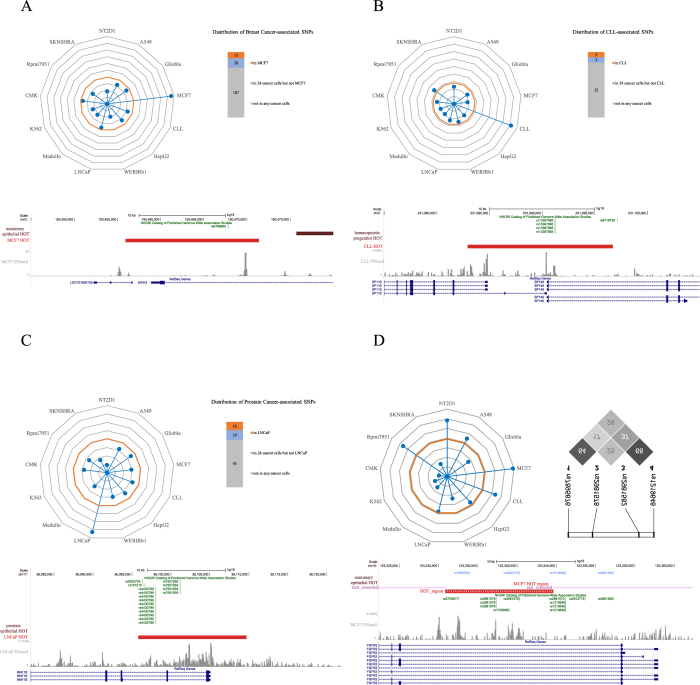
SNPs in cancer-specific HOT regions. (**A**) (Upper-left) Radar plot that shows the density of SNPs associated with breast cancer in HOT regions identified in 13 cancer cells. The centre of the plot is 0, and the coloured dot on the respective axis indicates the SNP density (SNP/MB) in the HOT regions of each cancer cell (the same as following radar plots). Yellow circles represent the percentage of breast cancer-associated SNPs in the noncoding GWAS catalogue (97/4,985, 1.94%). (Upper-right) Distribution of breast cancer-associated SNPs within HOT regions in MCF7 cells and 24 other cancer cells. (Bottom) A breast cancer-associated common variant rs6788895 is only located in a cancer-specific HOT region in the *SIAH2* locus. (**B**) (Upper-left) Radar plot that shows the density of SNPs associated with CLL in HOT regions identified in 13 cancer cells. Yellow circles represent the percentage of CLL-associated SNPs in the noncoding GWAS catalogue (26/4,985, 0.52%). (Upper-right) Distribution of CLL-associated SNPs within HOT regions in CLL cells and other 24 cancer cells. (Bottom) A CLL risk loci rs13397985 is only located in a cancer-specific HOT region surrounding the TSS of gene *SP140* and *SP110*. (**C**) (Upper-left) Radar plot that shows the density of SNPs associated with prostate cancer in HOT regions identified in 13 cancer cells. Yellow circles represent the percentage of prostate cancer-associated SNPs in the noncoding GWAS catalogue (97/4,985, 1.94%). (Upper-right) Distribution of prostate cancer-associated SNPs within HOT regions in LNCaP cells and 24 other cancer cells. (Bottom) Two prostate-cancer-associated SNPs (rs7501939 and rs4430796) are located in a cancer-specific HOT region in the *HNF1b* locus. (**D**) (Upper-left) Radar plot that shows the density of SNPs in strong LD (*r*^2^ > 0.8) with breast cancer-associated GWAS SNPs in HOT regions identified in 13 cancer cells. Yellow circles represent the percentage of breast cancer-associated SNPs in the noncoding GWAS catalogue (97/4,985, 1.94%). (Upper-right) The *r*^2^ values of LD analysis of the four intronic SNPs in the studied population of North India. (Bottom) Four breast-cancer-associated SNPs (rs2981582, rs1219648, rs2981578 and rs7895676) are found in or near a cancer-specific HOT region in *FGFR2* intron 2.

Inherited genetic susceptibility plays a key role in predisposition to common cancer^56^. GWAS have emerged as a powerful tool for discovering the common genetic susceptibility alleles that confer risk for cancer and has been used to identify a large number of cancer risk alleles^57^. To gain further insight into the disproportional enrichment of HOT regions for genetic variants and the bias of this enrichment toward cancer-specific cell types during tumour pathogenesis, we focused on several cancers in which GWAS SNPs or SNPs in strong LD with GWAS SNPs occur in HOT regions in cancer-specific cells and not in those of related healthy counterparts (Fig. 6). First, a common variant rs6788895, which is associated with ER-positive breast cancer in the *SIAH2* locus^58^, was only located in the MCF7 HOT region but not healthy counterparts (Fig. 6A). Second, the HOT region that harbours SNP rs13397985, which was reported as a CLL risk loci at 2q37.1 (*SP140*, *p*-value = 1.91 × 10^−20^)^59^, was found in CLL cells but not related healthy counterparts (Fig. 6B). Third, the HOT region that harbours prostate cancer-associated SNPs (rs7501939 and rs4430796) in hepatic nuclear factor 1 beta (*HNF1b*), which has been identified as a major risk gene for prostate cancer by several recent GWA studies^47^,^60^,^61^,^62^, was found in LNCaP cells but not related healthy cells (Fig. 6C). Fourth, we observed that a ~15-kb HOT region that harbours two SNPs (rs2981578 and rs7895676) was found in *FGFR2* intron 2 in breast cancer cells but not normal breast cells (Fig. 6D). These two SNPs were in LD with marker SNPs (rs2981582 and rs1219648)^63^, which were the most strongly associated marker SNPs in intron 2 of *FGFR2* that were highly associated with breast cancer^64^,^65^. Our findings further indicated that the disproportional enrichment of HOT regions for genetic variants is biased toward cancer-specific HOT regions.

### HOT regions in cancers

The effects of *cis*-regulatory elements on tumourigenesis have been substantially understudied compared with the large number studies regarding the variants in protein-coding genomes^5^,^6^,^7^,^8^,^9^. To investigate the functional roles of HOT regions in cancers, we identified HOT regions in 25 human cancer cells and their associated genes. A remarkable spectrum of known oncogene drivers in the cancer cell dataset had associated HOT regions, and these oncogenes were significantly enriched in HOT regions compared with LOT regions (Fig. S3A, χ^2^ test). These results suggest that HOT regions may be useful for identifying key oncogenes in specific cancers.

**Figure 7:**
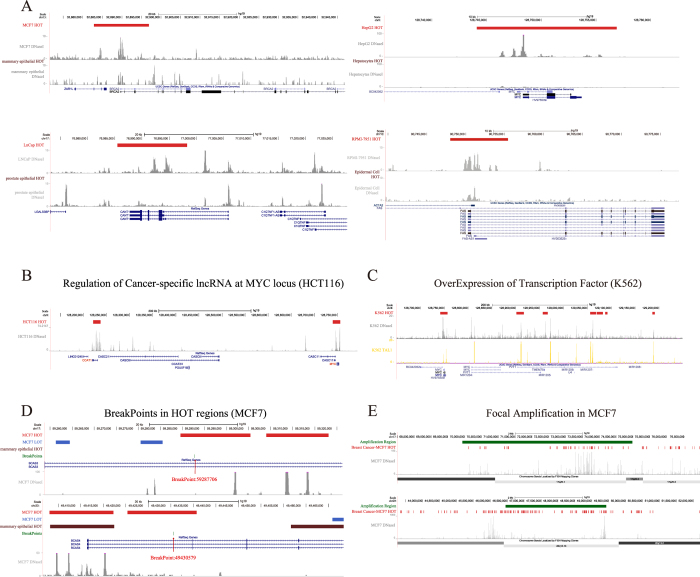
HOT regions in cancer. (**A**) Cancer cells acquire cancer-specific HOT regions. DNase-seq profiles are shown surrounding the oncogenes *BRCA2*, *MYC*, *CANT1* and *FAS* in breast cancer, hepatocellular carcinoma, prostate cancer, and malignant melanoma, respectively, as well as in their healthy counterparts. Cancer-specific HOT regions surround the TSS of the oncogene. (**B**) Colorectal cancer-specific CCAT1-L lncRNA regulates long-range chromatin interactions at the *MYC* locus. DNase-seq profiles are shown within the region between *CCAT1* and *MYC*; the HOT regions are found surrounding these two genes. (**C**) Tal1 binding is observed in HOT regions in K562 cells. The DNase-seq profile and ChIP-seq binding profile of TAL1 are coloured grey and yellow, respectively. (**D**) Two breakpoints of the fusion gene *BCAS3*-*BCAS4* are found in breast cancer-specific HOT regions. DNase-seq profiles surrounding these two breakpoints in MCF7 cell are shown. (**E**) Large HOT regions are observed at the site of focal amplification in breast cancer. See also Fig. S3 and Table S1.

Further analysis of the HOT regions in tumour cells and related healthy cells suggested that cancer cells acquire cancer-specific HOT regions at oncogene drivers during tumour pathogenesis (Fig. 7A). For example, a large HOT region was identified in the promoter surrounding the breast cancer susceptibility gene *BRCA2*^66^ in cancer cells but not in their healthy counterparts. Additionally, cancer-specific HOT regions were found surrounding the *c-MYC* gene, *CANT1* gene^67^, and *FAS* gene in hepatocellular carcinoma cells, prostate carcinoma cells, and malignant melanoma cells (Fig. 7A). Similar cancer-specific localisations of HOT regions surrounding oncogenes were observed for multiple additional cancers, including pancreatic cancer, prostate cancer, and chronic myelogenous leukaemia (Fig. S3B). These results indicate that cancer cells acquire cancer-specific HOT regions at key oncogenes in tumour cells but not in their healthy counterparts.

We reasoned that many mechanisms of carcinogenesis that frequently occur in cancers may account for the ability of cancer cells to acquire cancer-specific HOT regions. Indeed, we observed that cancer cells acquire cancer-specific HOT regions at key oncogenes through diverse mechanisms, including long-range chromatin looping, transcription factor overexpression, gene fusion, and focal amplification of lncRNA (Fig. 7B–E). For instance, a previously non-annotated lncRNA (*CCAT1*) located 515 kb upstream of the *MYC* locus plays an important role in *MYC* transcriptional regulation and promotes long-range chromatin looping. *CCAT1-L* interacts with CTCF and modulates the chromatin conformation in these loop regions^68^. In the human colon cancer cell line HCT116, two HOT regions were found in the *MYC* and *CCAT* genes, respectively (Fig. 7B). The aberrant expression of the oncogenic transcription factor *TAL1/SCL* can be detected in the majority of cases of human T cell acute lymphoblastic leukaemia (T-ALL)^69^,^70^. We observed that the overexpression of TAL1 in T-ALL is associated with HOT region formation in the *MYC* locus (Fig. 7C). A recent study demonstrated that the breakpoints in MCF-7 were not evenly distributed across the genome. Four rearrangement clusters emerged in 1p13.1–21.1, 3p14.1–p14.2, 17q22–q24.3, and 20q12–q13.33 and contained 43% of all MCF-7 somatic breakpoints^71^. We observed that HOT regions were significantly enriched within these four rearrangement clusters (binomial test, *p*-value = 7.66 × 10^−16^) (Fig. S3C). *BCAS3*-*BCAS4* gene fusion, which has previously been reported in breast carcinomas, was validated as the product of a fusion gene between BCAS4 and BCAS3, which resulted from amplification followed by a translocation event between the two loci chr20q13 and chr17q23^72^. Strikingly, both of the breakpoints associated with *BCAS3*-*BCAS4* gene fusion were found in the HOT regions in the cancer cells but not in their healthy counterparts (Fig. 7D). Comparative genomic hybridisation (CGH) on cDNA microarrays revealed hundreds of novel genes whose overexpression was attributable to gene amplification; these genes may provide insight into the clonal evolution and progression of breast cancer and highlight promising therapeutic targets^73^. Notably, we found that DNA amplification in breast cancer involved a large HOT region (Fig. 7E). Our findings suggest that cancer cells acquire cancer-specific HOT regions through diverse mechanisms of cancer pathogenesis.

## Discussion and Conclusions

Several recent studies have suggested that many noncoding variants associated with common diseases and traits are concentrated in regulatory DNA elements marked by DHSs^14^,^35^. However, the noncoding regulatory effects responsible for human disease and cancer biology remain poorly understood. To better characterise the *cis*-regulatory effects of noncoding variants, we performed a comprehensive analysis of the genetic variation in HOT regions, which were defined as TFBS-clustered regions with extremely high TFBS complexity in our recent study^34^.

First, we explored the dynamic changes in the genetic variation landscape along the hematopoietic lineage. Our findings demonstrated that GWAS variants in HOT regions undergo a substantial net decrease during haematopoiesis. Further enrichment analysis of disease- and trait-specific GWAS variants in HOT regions during hematopoietic development revealed development-specific enrichment of phenotype-associated variants within HOT regions. Taken together, our findings demonstrate cell- and development-specific localisation of GWAS variants within disease-relevant cell or tissue types and suggest a recurring connection among HOT regions, regulatory genotypes, and the risk for specific classes of diseases and traits. These results also highlight the potential for using a comprehensive map of HOT regions to illuminate associations between GWAS variants, diseases, HOT regions and their associated genes both within disease-relevant cell types and within definitive lineage derivatives.

Next, we examined the enrichment of genetic variants in both disease-relevant cells and cancer cells. We observed that genetic variants are disproportionally enriched in HOT regions compared with LOT regions in both disease-relevant and cancer cells, and the disproportional enrichment is not completely determined by the high concentration of TFBSs. Importantly, the enrichment is biased toward disease- or cancer-specific cells. Considering that HOT regions drive the expression of genes that control cell development and differentiation, our findings suggest that the altered expression of cell developmental genes may often contribute to human diseases and cancers. These results also suggest that the understanding of a cell- or tissue-specific regulatory role for GWAS variants in human diseases and cancers might be elucidated by investigation of disease- or cancer-specific HOT regions.

Finally, we investigated the functional roles of HOT regions in cancers because the noncoding genome has been disregarded in the search for causes of cancer, with the exception of a few isolated examples^5^,^6^,^7^,^8^,^9^. We identified HOT regions in 25 human cancer cells and their associated genes, and we observed that a remarkably broad spectrum of oncogenes that have been described in cancers^74^,^75^,^76^,^77^,^78^ were significantly enriched in HOT regions compared with LOT regions. Importantly, we observed that cancer cells generally acquired cancer-specific HOT regions at key oncogenes during tumour pathogenesis through a variety of mechanisms, including lncRNA long-range chromatin looping, transcription factor oncogene overexpression, gene fusion, and focal amplification. These findings suggest that HOT regions can provide biomarkers for cancer-specific pathologies that may be valuable for investigations into cancer biology, diagnosis, and therapy.

Collectively, our findings demonstrate a significantly strong association between HOT regions and disease-associated variants and cancers and reveal functional roles of HOT regions in human diseases and can cers. These findings provide a key step toward targeting the noncoding genome for clinical purposes. Future studies should focus on the molecular mechanisms of HOT regions in human diseases and cancers, especially in combination with the recently developed CRISPR/Cas9 system. Two recently published studies utilised a CRISPR/Cas9 genome editing strategy to reveal the functional roles of super-enhancers^79^,^80^. Gröschel *et al*. applied functional genomics and genome editing to characterise chromosome 3q-rearrangements of a super-enhancer in primary AML samples and human cell lines^79^. Another study demonstrated that a distal super-enhancer was required for the maintenance of the pluripotency transcription program in mouse ESCs using a double-CRISPR genome editing technique^80^. Thus, the systematic association of HOT regions with human diseases and cancers can be further validated using the efficient CRISPR/Cas9 genome editing strategy. This study will serve as a paradigm for investigations of functional annotation of the noncoding genome.

## Materials and Methods

### Data sets

The DNaseI Hypersensitivity by Digital DNaseI data were obtained from the Duke and UW ENCODE groups. Gene annotations were obtained from the GENCODE data (V15). All of these data were provided through the ENCODE Project^10^,^11^, and use of the data strictly adheres to the ENCODE Consortium Data Release Policy.

### Identification of TFBS-clustered regions and HOT regions

In our recent study^34^, we identified TFBS-clustered regions using Gaussian kernel density estimation (bandwidth 3 kb) across the binding profiles of 542 TFs and defined a “TFBS complexity” score based on the number and proximity of contributing TFBSs for each TFBS-clustered region. To identify HOT regions, we first ranked all the TFBS-clustered regions in a cell type and plotted them in order of increasing TFBS complexity. This plot revealed a clear point in the distribution of the TFBS-clustered regions at which the complexity signal began to increase rapidly. To geometrically define this point, we first scaled the data such that the *x* and *y* axes were from 0-1. We then found the *x*-axis point for which a line with a slope of 1 was tangent to the curve. We defined the TFBS-clustered regions above this point to be HOT regions and the TFBS-clustered regions below this point to be LOT regions. The pipeline for identifying HOT or LOT regions was applied uniformly to datasets from 349 samples, including 154 cell types studied under the ENCODE Project^10^,^11^.

### Characterisation of disease-associated GWAS SNPs in HOT regions

Disease-associated SNPs were downloaded from the NHGRI database of GWAS on Feb 18, 2014; at this time, the database contained 15,698 entries/rows. Because SNPs that are reproducibly associated with a trait have been suggested to have a higher likelihood of being causative^14^, we only considered SNPs that contained a dbSNP identifier and were found to be associated with a trait by at least two independent studies. A total of 6,955 SNP trait or disease associations were used in Fig. S1B. A total of 6,369 noncoding SNP-trait associations were used for Figs 1, 2 and 4, 5. A total of 4,985 unique SNPs located outside of the coding regions were used for Figs 1,6, and S1A.

### GWAS SNPs localise in development-specific HOT regions along the hematopoietic lineage

SNPs that map in “lost” HOT regions along the hematopoietic lineage were defined as those SNPs located in HOT regions that belong to a progenitor cell type that were not found within a more differentiated cell type. Conversely, SNPs that map in “gained” HOT regions along the hematopoietic path were defined as SNPs located in HOT regions that belong to a more differentiated cell type but not its progenitor. SNPs that map in “shared” HOT regions within the hematopoietic lineage were defined as SNPs located in HOT regions that belong to a more differentiated cell type and its progenitor.

For each developmental stage along the hematopoietic lineage, we computed the enrichment of GWAS SNPs from particular diseases or traits in development-specific HOT regions (Fig. 2B) by dividing the proportion of GWAS SNPs in development-specific HOT regions by the overall proportion of GWAS SNPs in development-specific HOT regions (44% for ESCs, 24% for Hemat, 63% for B cells, and 41% for T cells). The enrichment is reported as the percentage enrichment or depletion. The individual significance levels of these enrichments were computed using the binomial distribution *b*(*x*; *n*, *p*), setting the parameter *x* to the number of GWAS SNPs of a given disease or trait in development-specific HOT regions, *n* to the number of GWAS SNPs for the disease or trait, and *p* to 0.44, 0.24, 0.63, 0.41 for ESC, hemat, B cells, or T cells, respectively. To compensate for the overall enrichment or depletion of disease categories in HOT regions in general, GWAS SNPs not located in any HOT regions were excluded.

### Enrichment of SNPs in HOT and LOT regions

The densities of trait-associated noncoding SNPs in HOT and LOT regions of individual cell and tissue samples were calculated by first counting the number of SNPs found in these regions. Then, the numbers were divided by the number of base pairs in the HOT and LOT regions of the genome in these cells and multiplied by 1 million to obtain a SNP/MB value (Figs. 3, 4, 5, 6).

To explore the association of genetic variants with HOT regions in human diseases and cancers, we calculated the SNP densities in HOT/LOT regions identified in both disease-relevant cells and cancer cells (Fig 3A,B). To investigate the cause of the disproportional enrichment of HOT regions for genetic variants compared with LOT regions, we calculated the SNP densities within the genome-wide TFBSs in the human genome and TFBSs that map to corresponding HOT/LOT regions across 154 ENCODE cell lines (Fig. 3C,D).

Additionally, we used CEU population genotype data from the 1000 Genomes Project^81^ to compute the LD measurement *r*^2^ between GWAS SNPs and SNPs in the HOT regions located near them. We illustrated LD plots in Figs 5 and 6D using Haploview^82^. We computed *r*^2^ between each such GWAS SNP lying outside of a HOT region and every SNP within a 125 kb radius of a HOT region. Then, we calculated the density of trait-associated noncoding SNPs achieving *r*^2^ > 0.8 with a SNP lying within a HOT region and a LOT region within a 125 kb radius in individual cell and tissue samples (Fig. 5). For Fig. 6D, the *r*^2^ values of LD analysis of the four intronic SNPs in the studied population of North India were obtained from a recent study^63^.

The significance of the number of SNPs in HOT regions was calculated using a permutation test. HOT regions were randomly shifted on the chromosome of origin 10,000 times. The number of SNPs that fell into these shifted regions was counted. No iteration of this test resulted in the same or a greater number of trait-associated SNPs in HOT regions.

### Association of HOT regions with cancers

A total of 522 proto-oncogenes were obtained from COSMIC (Catalogue of Somatic Mutations in Cancer)^75^,^76^.

### Accession numbers

The identified HOT regions across human cell and tissue types have been deposited with the Gene Expression Omnibus under the accession ID GSE54296.

## Additional Information

**How to cite this article**: Li, H. *et al*. Functional annotation of HOT regions in the human genome: implications for human disease and cancer. *Sci. Rep*. **5**, 11633; doi: 10.1038/srep11633 (2015).

## Supplementary Material

Supplementary Information
