# Supplementary material for: Functional annotation of HOT regions in the human genome: implications for human disease and cancer
Source: Sci Rep. 2015 Jun 26;5:11633. doi: 10.1038/srep11633 (PMC4481521; doi:10.1038/srep11633)
Supplement: Supplementary Information [file srep11633-s1.pdf]

Supplementary materials for

Functional annotation of HOT regions in the human genome: implications for human disease and cancer

Hao Li[1], Hebing Chen[1], Feng Liu[1], Chao Ren[1], Shengqi Wang[1], Xiaochen Bo[1*], Wenjie Shu[1*]

[1]Department of Biotechnology, Beijing Institute of Radiation Medicine, Beijing 100850, China

[*]Corresponding author. To whom correspondence should be addressed. Tel & Fax: +86 10 68210077 66932211; Email: shuwj@bmi.ac.cn. Correspondence may also be addressed to: boxc@bmi.ac.cn.

Supplementary figures

Supplementary Figure S1. Catalogue of SNPs linked to phenotypic traits and diseases in GWASs, related to Figure 1

(A) Proportions of noncoding GWAS SNPs localised within HOT regions (blue), in strong LD ($r^2 > 0.8$) with a SNP in a HOT region (red), or neither (green). Note that 84% of the GWAS SNPs are either within HOT regions or in strong LD with HOT regions. (B) Pie chart that shows the percentages of SNPs associated with the highlighted classes of traits and diseases. (C) The SNP enrichment values of noncoding SNPs linked to the highlighted traits and diseases in the union of HOT regions and LOT regions in 57 human cell and tissue samples.

Supplementary Figure S2. GWAS SNPs in disease- and trait-specific HOT regions, related to Figure 4

(A) (Upper) Bar plots that show the density (SNP/MB sequence) of trait-associated noncoding SNPs linked to ventricular conduction in the HOT and LOT region domains identified in 16 human cell and tissue types. (Middle) List of genes associated with ventricular conduction SNP-containing HOT regions in heart cells. (Bottom) DNase-seq profiles at the *SCN10A* locus in HCM cell. The positions of the ventricular conduction SNPs are highlighted with red lines, the HOT regions are highlighted with red bars, and the LOT regions are highlighted with blue bars above the binding profile. (B) (Upper) Bar plots that show the density (SNP/MB sequence) of trait-associated

noncoding SNPs linked to rheumatoid arthritis in the HOT and LOT region domains identified in 16 human cell and tissue types. (Middle) List of genes associated with rheumatoid arthritis SNP-containing HOT regions in lymphoid cells. (Bottom) DNase-seq profile surrounding rheumatoid arthritis SNP rs657075 (red line). The HOT regions are highlighted with red bars, and the LOT regions are highlighted with blue bars above the binding profile. (C) (Upper) Bar plots that show the density (SNP/MB sequence) of trait-associated noncoding SNPs linked to celiac disease in the HOT and LOT region domains identified in 16 human cell and tissue types. (Middle) List of genes associated with celiac disease SNP-containing HOT regions in Th cells. (Bottom) DNase-seq profile in Th cells. The positions of celiac disease SNPs are highlighted with red lines, the HOT regions are highlighted with red bars, and the LOT regions are highlighted with blue bars above the binding profile.

Supplementary Figure S3. HOT regions in cancers, related to Figure 7

(A) Enrichment of 522 oncogenes associated with HOT regions and LOT regions in 25 cancer cell lines. (B) DNase-seq profiles are shown surrounding the oncogene in selected cancers and their healthy counterparts. Cancer-specific HOT regions are found surrounding the TSS of oncogenes. (C) Percentage of cancer and normal HOT and LOT regions in breakpoint clusters.

Supplementary tables

Table S1. 154 Cell lines used in this study

154 Cell lines used in this study.

Table S2. SNPs in HOT, LOT and TFBS-clustered regions, related to Figure 1

Summary of trait-associated SNPs in the union of the HOT and LOT regions in 154 human cell lines. The percentage of the 4,985 trait-associated noncoding SNPs located in these regions is displayed. The percentage of the genome (3.4 billion bases) covered by the union of these regions in the 154 human cell and tissue types is also displayed. The SNP enrichment is defined as the percentage of SNPs contained in the percentage of the genome covered by these regions.

S1A

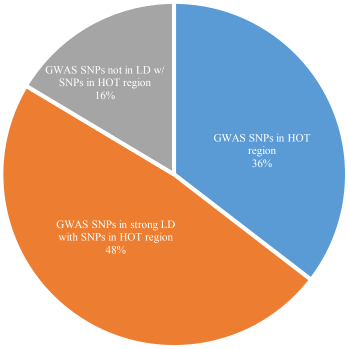

S1B

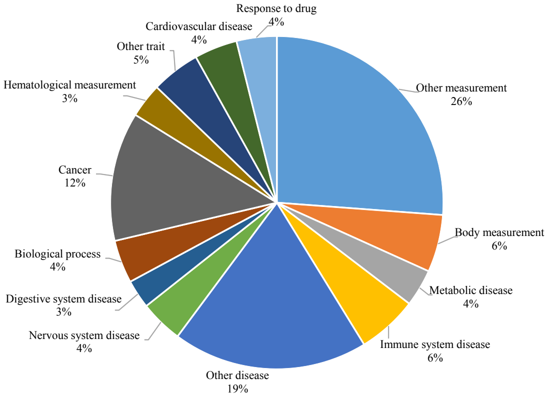

S1C

Trait enrichment

Trait	HOT region	LOT region
Multiple sclerosis	4.21	1.60
Systemic sclerosis	4.21	1.60
Chronic lymphocytic leukemia	4.14	1.59
Type 1 diabetes	3.75	1.55
Celiac disease	3.70	1.61
Rheumatoid arthritis	3.23	1.51
Inflammatory bowel disease	3.16	1.55
Colorectal cancer	3.11	1.61
Crohn’s disease	3.09	1.55
Systemic lupus erythematosus	3.05	1.56

S2A

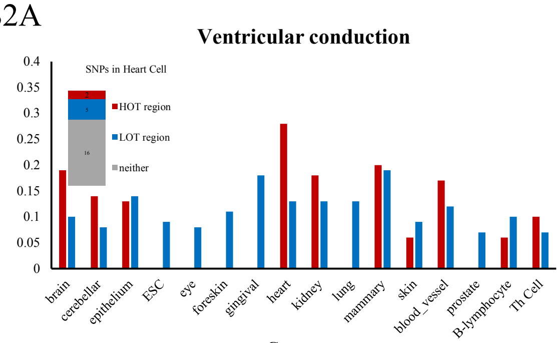

Gene				
KLF12	PRKCA	SETBP1	CRIM1	SCN10A
TKT				

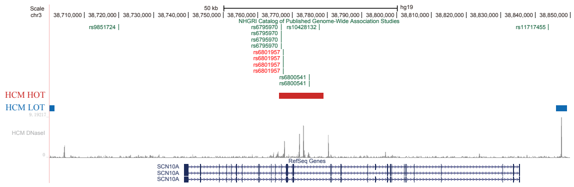

S2B

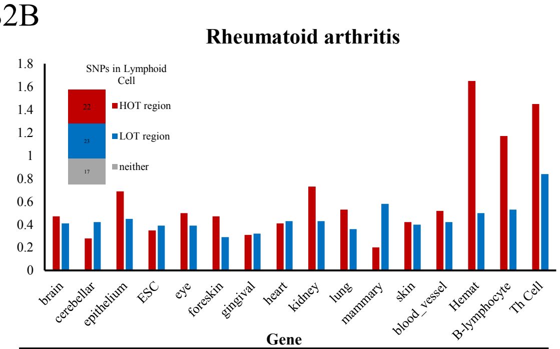

Gene				
MMEL1	CD247	IL2RA	ARID5B	PDE2A
KIF5A	GCH1	PLD4	PRKCB	REL
SPRED2	STAT4	CTLA4	CD40	UBASH3A
ARHGEF3	KIAA1109	C5orf30	APOM	CCR6
CDK6	TNPO3	BLK	TRAF1	

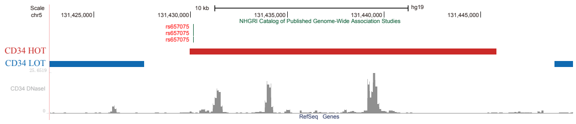

S2C

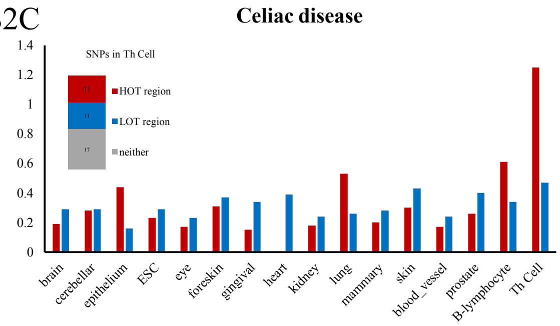

Gene				
CD247	ZMIZ1	ICOS	ICOSLG	ARHGAP31
LPP	KIAA1109	HLA-DQA1	BACH2	TAGAP
ELMO1				

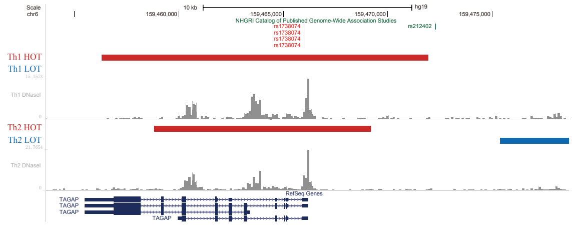

S3A

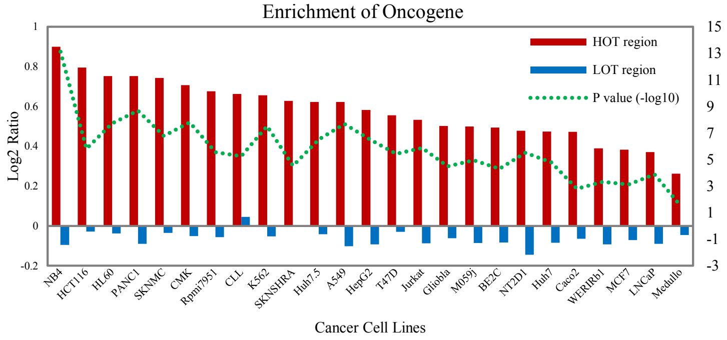

S3B

Pancreatic cancer

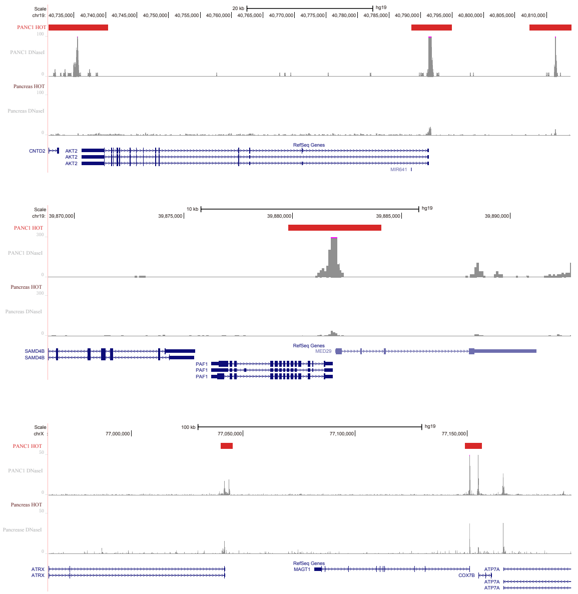

Prostate cancer

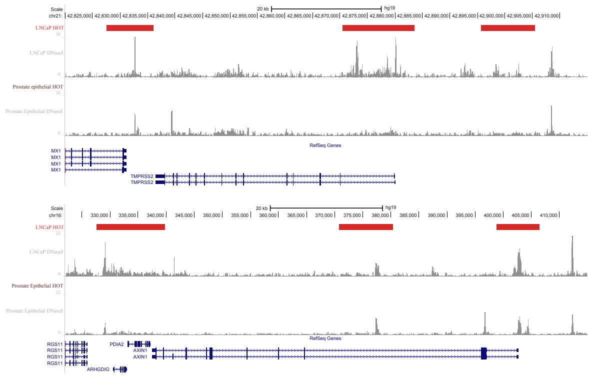

Leukemia

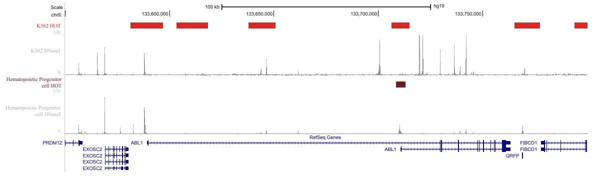

S3C

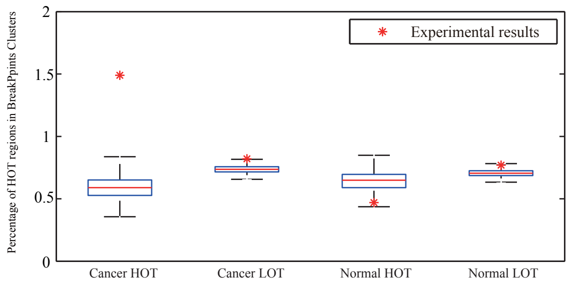

Table S1. 154 Cell Lines Used in this Study

Use as Cancer Cell	Use as Normal Cell		Others	
Duke_CLL	Duke_GM12891	Uw_HCF	Cd4naivewb11970640_UW	Lhcnm2_UW
Duke_Gliobla	Duke_GM12892	Uw_HCM	Cd4naivewb78495824_UW	Msc_UW
Duke_Huh7	Duke_GM18507	Uw_HConF	Duke_8988T	Nhbera_UW
Duke_Huh7.5	Duke_GM19238	Uw_HEEpiC	Duke_AoSMC	Th17_UW
Duke_Medullo	Duke_GM19239	Uw_HFF	Duke_Chorion	Tregwb83319432_UW
Duke_T47D	Duke_GM19240	Uw_HGF	Duke_Fibrobl	Uw_AG04450
M059j_UW	Duke_H9ES	Uw_HMF	Duke_Fibrobl.overlap	Uw_CD20
Rpmi7951_UW	Duke_HPDE6E6E7	Uw_HMVECdAd	Duke_FibroP	Uw_GM06990
Uw_BE2C	Duke_RWPE1	Uw_HMVECdBlAd	Duke_HeLaS3IFNa4h	Uw_GM12864
Uw_Caco2	Hbvp_UW	Uw_HMVECdBlNeo	Duke_Hepatocytes	Uw_HAEpiC
Uw_CMK	Hbvsmc_UW	Uw_HMVECdLyAd	Duke_HSMMemb	Uw_HAsp
Uw_HCT116	Th1_UW	Uw_HMVECdLyNeo	Duke_HTR8svn	Uw_HBMEC
Uw_HL60	Th1wb33676984_UW	Uw_HMVECdNeo	Duke_iPS	Uw_HCFaa
Uw_Jurkat	Th1wb54553204_UW	Uw_HMVECLBl	Duke_IshikawaEstradiol	Uw_HCPEpiC
Uw_NB4	Th2_UW	Uw_HMVECLLy	Duke_IshikawaTamoxifen	Uw_HFFMyc
Uw_NT2D1	Th2wb33676984_UW	Uw_HPAEC	Duke_LNCaPAndrogen	Uw_HIPEpiC
Uw_PANC1	Th2wb54553204_UW	Uw_HPAF	Duke_MCF7Hypoxia	Uw_HNPCEpiC
Uw_SKNMC	Tregwb78495824_UW	Uw_HPdLF	Duke_Melano	Uw_HRCEpiC
Uw_SKNSHRA	Uw_AG04449	Uw_HPF	Duke_Myometr	Uw_HRE
Uw_WERIRb1	Uw_AG09309	Uw_HRGEC	Duke_Osteobl	Uw_HVMF
UWDuke_A549	Uw_AG09319	Uw_HRPEpiC	Duke_PanIsletD	Uw_MonocytesCD14RO01746
UWDuke_HepG2	Uw_AG10803	Uw_NHA	Duke_PanIslets	Uw_NHLF
UWDuke_K562	Uw_AoAF	Uw_NHDFAd	Duke_pHTE	Uw_RPTEC
UWDuke_LNCaP	Uw_BJ	Uw_NHDFneo	Duke_ProgFib	Uw_SKMC
UWDuke_MCF7	Uw_CD34Mobilized	Uw_PrEC	Duke_Stellate	Uw_Th2
	Uw_GM12865	Uw_SAEC	Duke_Th0	Uw_WI38
	Uw_H7hESC	UWDuke_GM12878	Duke_Urothelia	Uw_WI38TamoxifenTamoxifen
	Uw_HAc	UWDuke_H1hESC	Duke_UrotheliaUT189	UWDuke_HeLaS3
	Uw_HAh		Gm04503_UW	UWDuke_HMEC
			Gm04504_UW	UWDuke_HSMM
			H7es_UW	UWDuke_HSMMtube
			H7esDiffa14d_UW	UWDuke_HUVEC
			H7esDiffa2d_UW	UWDuke_NHEK
			H7esDiffa5d_UW	UWDuke_Th1
			H7esDiffa9d_UW	Hs27a_UW
			Hmec_UW	Hs5_UW

Table S2. SNPs in HOT, LOT and TFBS-clustered regions, related to figure 1B

	HOT region	LOT region	TFBS-clusters
Trait-associated SNPs	35.45%	85.18%	86.48%
% genome contrained	15.27%	58.08%	58.60%
SNP enrichment	2.32	1.47	1.48
